# The role of communication inequality in mediating the impacts of socioecological and socioeconomic disparities on HIV/AIDS knowledge and risk perception

**DOI:** 10.1186/1475-9276-13-16

**Published:** 2014-02-10

**Authors:** Mesfin Awoke Bekalu, Steven Eggermont

**Affiliations:** 1 Bahir Dar University, Bahir Dar, Ethiopia; 2 University of Leuven, Leuven, Belgium

**Keywords:** Communication inequality, Socioecological factors, Socioeconomic factors, HIV/AIDS

## Abstract

**Introduction:**

Although the link between social factors and health-related outcomes has long been widely acknowledged, the mechanisms characterizing this link are relatively less known and remain a subject of continued investigation across disciplines. In this study, drawing on the structural influence model of health communication, the hypothesis that differences in concern about and information needs on HIV/AIDS, HIV/AIDS-related media use, and perceived salience of HIV/AIDS-related information, characterized as communication inequality, can at least partially mediate the impacts of socioecological (urban vs. rural) and socioeconomic (education) disparities on inequalities in HIV/AIDS knowledge and risk perception was tested.

**Methods:**

Data were collected from a random sample of 986 urban and rural respondents in northwest Ethiopia. Structural equation modeling, using the maximum likelihood method, was used to test the mediation models.

**Results:**

The models showed an adequate fit of the data and hence supported the hypothesis that communication inequality can at least partially explain the causal mechanism linking socioeconomic and socioecological factors with HIV/AIDS knowledge and risk perception. Both urbanity versus rurality and education were found to have significant mediated effects on HIV/AIDS knowledge (urbanity vs. rurality: *β* = 0.28, *p* = .001; education: *β* = 0.08, *p* = .001) and HIV/AIDS risk perception (urbanity vs. rurality: *β* = 0.30, *p* = .001; education: *β* = 0.09, *p* = .001).

**Conclusions:**

It was concluded that communication inequality might form part of the socioecologically and socioeconomically embedded processes that affect HIV/AIDS-related outcomes. The findings suggest that the media and message effects that are related to HIV/AIDS behavior change communication can be viewed from a structural perspective that moves beyond the more reductionist behavioral approaches upon which most present-day HIV/AIDS communication campaigns seem to be based.

## Introduction

The fact that social factors affect health-related outcomes directly or indirectly has garnered considerable evidence over the past several decades. Most of the factors referred to as the social determinants of health might well be seen in terms of and/or subsumed by the three most notable and widely studied factors: socioeconomic status (SES), social capital, and socioecological factors. The role of social factors in health has become increasingly apparent in recent years; however, our understanding of the mechanisms through which such factors exert influences is relatively incomplete [1,2] and remains a controversial subject [3]. Research and theory in public health and the social sciences posit that there are multiple potential pathways through which social factors determine health outcomes and urge that analyses address macroeconomic and macrosocial factors, immediate social environments, individual psychological and behavioral factors, and biological predispositions and processes [4,5].

In a review synthesizing knowledge about SES and health, Adler and Ostrove [4] observed an increase in the number of studies purporting to address the mechanisms by which SES affects health. This increasing body of research has identified several mediating factors, such as behavior, lifestyle, environmental exposure, health care, biological determinants, and chronic stress [6,7]. For instance, research has pointed toward specific behavioral factors, such as smoking, physical activity, and breakfast consumption, as mechanisms that can partly explain the relationship between SES and self-rated health [8]. The relationship has also been shown to be mediated by stressors and psychosocial resources [9]. In addition, research has isolated individual inability to understand health information and/or communicate well with health care providers as important mediating factors, and in most cases, these factors are associated with low SES [2]. Indeed, in a series of studies, Viswanath and colleagues showed that communication inequality is an important factor that can at least partially explain the link between social determinants and health outcomes [10-13]. The present study seeks to extend and/or add to this body of research by assessing the role of HIV/AIDS communication inequality in mediating the impact of social factors on HIV/AIDS-related outcomes among people who are highly affected by the epidemic.

### Communication inequality

**Figure 1:**
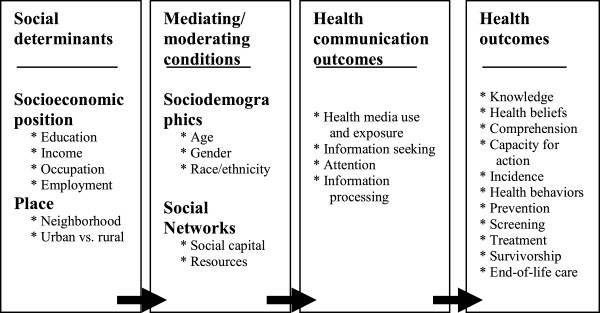
**Structural influence model (Viswanath et al. **[15]**).**

Despite its long history in the literature [14], the term *communication inequality* seems to have acquired currency in the realm of health communication in recent years. Communication inequality “may be defined as differences in the generation, manipulation, and distribution of information among social groups; and differences in (a) access and use, (b) attention, (c) retention, and (d) capacity to act on relevant information among individuals” ( [13], p. 242). As elaborated in the structural influence model (SIM) of health communication [15] (Figure 1), the motivation for, access to, and use of health information and/or health-related media could at least partially explain the relationship between social determinants and health outcomes. The SIM heavily influenced the present study; its premise is that “audiences attend and react to mediated content based on their structural location in the environment and the social roles they play at any given time” ( [13], p. 244). This model attempts to encapsulate a body of work that views media and message effects from a more structural approach by moving beyond the more reductionist view of effects that has characterized the field of media studies for decades. The model contends that structural antecedents (such as SES and geography) determine both the information environment and the resources that are available for consumption and suggests that communication may have a role in linking social determinants with health outcomes [15].

Several studies have provided empirical support for this model. For example, in a nationally representative cross-sectional study in the United States, Viswanath and Ackerson [12] found that social determinants that have long been linked to health outcomes, such as race, ethnicity, language, and social class, are also strongly related to health-related and, more specifically, to cancer-related media use. In Japan, Ishikawa, Nishiuchi, Hayashi, and Viswanath [16] found similar results. Health communication outcomes, such as seeking health information, self-efficacy in seeking health information, and exposure to and trust in health information from different media, were patterned by SES, which in turn has long been linked to disparities in health outcomes. Inequality in the access to, trust in, and use of health information is not limited to the information obtained from a particular source; rather, it spans all available information sources, including the Internet. Despite the Internet’s potential and increasing usefulness as a rich source of health information [17], studies have found that the Internet is more likely to be utilized by people who are better educated and have higher incomes and by younger, employed, and urban residents; these demographics present the possibility that the differences in Internet access and use could exacerbate health disparities between population subgroups [18-20].

Overall, the evidence suggesting that communication inequality might be a causal mechanism linking social determinants with health outcomes seems to be increasing. The evidence thus far, however, appears to be limited in at least two respects: the study context and type of health problem addressed. To date, nearly all of the studies were conducted in the context of developed nations in general and in the United States in particular, and the great majority of these studies focused on cancer [12,16]. The evidence provided by this body of research is likely to be relevant in other context. However, the importance of context for causal explanations [21] makes it imperative that further studies be conducted in various contexts. The main objective of the present study is therefore to elucidate the role of communication in explaining how the outcomes of a widely prevalent public health threat with enormous impacts could vary across population subgroups in a context that has scarcely been addressed by previous studies. More specifically, we focus on HIV/AIDS in a high-prevalence context (Ethiopia) and aim to determine whether knowledge about and the risk perception of the pandemic vary across socioeconomic and urban versus rural groups. Additionally, we examined whether such variations could be explained, at least in part, by HIV/AIDS communication inequalities. We anticipate that the study will contribute to the growing body of work targeting the controversies surrounding the link between social factors and health-related outcomes. More specifically, we expect that this endeavor will offer contextual breadth and relevant insights into the current understanding of the role of communication inequality and the effects of media and messages from a structural perspective.

### Hypothesis

In the arena of HIV/AIDS prevention in sub-Saharan Africa, both education and area of residence (urban vs. rural) play important roles. Previous research in the region has shown that people with better educations generally tend to have better HIV/AIDS-related outcomes (knowledge, beliefs and behaviors) compared with less-educated individuals [22-24]. Such variations also hold across urban versus rural groups [25,26]. Indeed, the area of residence in general and urban versus rural residences in particular have long been widely implicated as health determinants [27-29]. The urban–rural divide in sub-Saharan Africa can be seen against two useful backgrounds: epidemiological and socioecological. According to UNAIDS [30], in this region, only Senegal has a higher rural HIV prevalence. In the remaining countries, the prevalence rates are generally higher in urban rather than in rural areas, and Ethiopia has the most pronounced difference: urbanites in Ethiopia are eight times more likely to be HIV-infected than are rural residents. Moreover, in sub-Saharan Africa, the urban and rural contexts exhibit myriad socioecological differences (e.g., in cultural norms, social networks, health facilities and infrastructures, and information and communication technologies) [31-34].

**Figure 2:**
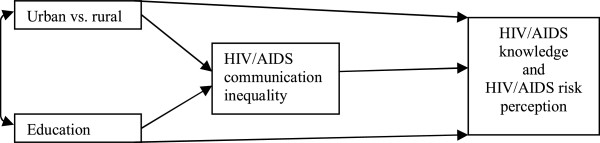
Hypothesized model.

While the impacts of education and area of residence on HIV/AIDS-related outcomes appear to be well documented, the evidence addressing the mechanisms through which such impacts could be exerted is limited. In an attempt to respond to this paucity of information and to contribute to current understanding of the role of communication inequality, we hypothesized that HIV/AIDS-related communication might be patterned by education level and area of residence and might thereby be a pathway through which these factors affect HIV/AIDS-related outcomes. To test our hypothesis, we developed two structural models in which HIV/AIDS communication inequality was expected to somewhat mediate the effects of area of residence and education on HIV/AIDS knowledge and HIV/AIDS risk perception (see Figure 2).

The SIM (Figure 1) posits that health communication inequality can be seen in terms of inequalities in health information-seeking behavior, use, and/or exposure to health-related media content, motivation for and/or attention to health-related information, and information-processing capacities [15]. In line with this proposition, in this study, communication inequality was represented by and/or comprised of three specific variables: (1) concern about and information needs related to HIV/AIDS (2) HIV/AIDS-related media use, and (3) the perceived salience of the HIV/AIDS-related information.

## Methods

### Sample and procedure

A household survey was conducted between March and June 2011 in two urban towns (Bahir Dar and Debre Markos) and two rural villages (Jajirab and Kurfa) in northwest Ethiopia. Data were gathered from 986 respondents (497 urban and 489 rural; 46.5% male, 53.5% female) aged 15 to 34 years (*M* = 25.4, *SD* = 6.2). In sub-Saharan Africa, HIV is mainly transmitted via heterosexual intercourse [35,36]; therefore, sexually active individuals (i.e., the youth) are the most vulnerable members of society, which supported our decision to sample the 15- to 34-year-old age group.

Randomly selected households were visited in the four study areas, and individuals who were available during the visit participated if they were eligible (in terms of age) and willing to participate and were the same gender as the enumerators. College-educated enumerators were recruited, trained, and deployed to administer the questionnaire by reading it aloud to all of the participants, as the majority of the rural respondents were illiterate, and we wished to ensure high response and return rates. We attempted to address a possible social desirability bias by selecting enumerators who were not known locally but were familiar with the local dialect and customs and by using same-gender enumerator-respondent combinations [37]. The study proposal and its working protocols were submitted to Bahir Dar University, the nearest academic and research institution in the region and the location where the fieldwork occurred; the university provided ethical approval.

### Measures

#### HIV/AIDS-related knowledge

A detailed measure of HIV/AIDS knowledge was prepared based on previous measures [38-40]. The measure comprised 40 items covering seven content areas: awareness of HIV/AIDS (α = .70), transmission modes (α = .94), transmission prevention (α = .87), high-risk behaviors (α = .87), the appearance of people living with HIV/AIDS (PLWHA) (α = .80), the existence of a cure (α = .81), and mortality (α = .87). The items were presented with three response options: *Yes*, *No*, and *Don’t Know*. The most commonly accentuated problem in most knowledge measures is the confounding of knowledge with belief [40,41]. Often, there are situations in which individuals might “know” the facts that are presented by experts but do not believe them. Hence, Zimet [40] suggests that one way of measuring knowledge per se without confounding it with belief is to begin knowledge measurement items with the phrase “Do most experts say…”. Therefore, the 40 items that were used to measure HIV/AIDS-related knowledge in the present study all began with “Do most experts say …”. The individual scores were calculated from a total of 40 points (*M* = 22.67, *SD* = 13.14).

#### HIV/AIDS risk perception

This variable measured the participants’ perceptions of severity and susceptibility. The perception of severity was measured by three items: (1) Getting HIV is a sure death sentence; (2) Getting HIV/AIDS is nothing special and is like any other disease (inversely scored); and (3) Getting HIV/AIDS is the worst thing that could happen to me. The scale had a good internal consistency (α = .86; *M* = 3.22, *SD* = 0.83). The perception of susceptibility was measured by four items: (1) I am at risk for getting HIV/AIDS; (2) I may have had sex with someone who is at risk for HIV; (3) My sexual experiences do not put me at risk for HIV (inversely scored); and (4) There is a possibility that I have HIV. The scale had good reliability (α = .96; *M* = 3.10; *SD* = 1.20). The HIV/AIDS risk perception was calculated by averaging the two measures (*M* = 3.16, *SD* = 0.80).

#### HIV/AIDS-related media use

This variable refers to an individual’s deliberate and/or nondeliberate use of mass media (i.e., print, radio, and television) for HIV/AIDS-related information over the previous month. Three items with seven-point response categories were used: In the last month, how often, on average, did you (1) read HIV/AIDS-related articles/stories in newspapers, pamphlets, or brochures?; (2) listen to HIV/AIDS-related messages on radio? (3) watch HIV/AIDS-related messages/shows on television? The response categories were 1 = Not at all; 2 = Once a week; 3 = 2–3 times a week; 4 = 4–5 times a week; 5 = 6–7 times a week; 6 = 8–9 times a week; and 7 = 10 or more times a week. The overall HIV/AIDS-related media use was calculated by averaging the three items (*M* = 1.24; *SD* = 0.32).

#### Concern about and information needs related to HIV/AIDS

This variable was measured using the following items: (1) HIV/AIDS is the disease I am currently most concerned about and would like to get information about; (2) HIV/AIDS is the disease that concerns most people in my community and they would like to obtain more information about it; and (3) HIV/AIDS is the most common cause of poor health and death in my community. The respondents were asked to indicate their agreement on a five-point scale that ranged from 1 (strongly disagree) to 5 (strongly agree). The measure had good internal consistency (α = .81, *M* = 3.76, *SD* = 0.76).

#### Perceived salience of HIV/AIDS-related information

This variable was assessed using a measure that consisted of three items: (1) I feel that the HIV/AIDS-related information that is being disseminated via different channels is relevant to me, (2) I feel that the HIV/AIDS-related information that is being disseminated via different channels is relevant to residents of my community, and (3) I feel that the HIV/AIDS-related information that is being disseminated via different channels is applicable to my current situation. The response categories ranged from 1 (strongly disagree) to 5 (strongly agree). The scale had good internal consistency (α = .90, *M* = 3.13, *SD* = 1.05).

#### Area of residence

This variable was measured as part of the demographic data. The participants were asked to identify themselves as either urban or rural. Prior to the data collection, the study areas were designated as urban and rural. The designation was based on relevant literature [42,43] and the criteria used by the Ethiopian Central Statistical Agency [44]. Accordingly, the urban samples had to have (1) a population of more than 1,000 people who were primarily engaged in nonagricultural activities and (2) a considerable number of commercial and manufacturing establishments and public places, such as hotels, bars, and pensions. The rural samples had to have (1) a population of less than 1,000 people who were primarily engaged in agricultural activities and (2) zero or few commercial and manufacturing establishments and public places. Urbanity was coded as 1, and rurality was coded as 0 (urban = 50.4%; rural = 49.6%).

#### Education

This variable was measured as part of the respondents’ demographics and coded as 1 = no education (46.3%), 2 = primary (32.0%), 3 = secondary (13.4%), and 4 = tertiary (8.2%).

### Data analysis

The analysis began by calculating descriptives and zero-order correlations to create an overview of the relationships between the variables. To obtain preliminary evidence about whether urbanity versus rurality and education affected HIV/AIDS knowledge and HIV/AIDS risk perception, two linear regression models were performed, with demographics (age and gender) included as controls. The hypothesized model was then tested with structural equation modeling (Amos) using the maximum likelihood method. Two models were tested: one with HIV/AIDS knowledge as an endogenous variable and the other with HIV/AIDS risk perception as an endogenous variable. As indicated in the model, communication inequality (represented by HIV/AIDS concern and information needs, HIV/AIDS-related media use, and the perceived salience of HIV/AIDS-related information) has been hypothesized to mediate the relationships only partially. To test whether there was full or partial mediation, two constrained models were tested, and their fit indices were compared with those of the unconstrained models.

Following the recommendations of Bollen and Long [45], the models were assessed using several fit indices. These indices included the chi-squared to degree of freedom ratio (CMIN/DF), which must be < 5.0; the comparative fit indices (CFI), which must be ≥ .95; the root mean square error of approximation (RMSEA), which should be < .08; and the goodness-of-fit statistic (GFI) and the adjusted goodness-of-fit statistic (AGFI), which must be > .95 and .90, respectively.

Further, following Aish and Jöreskog [46], insignificant paths were removed, and the fit indices of the models before and after the removal were compared. In addition to estimates of the total mediation (indirect) effects, we calculated the individual indirect effects of the three components of HIV/AIDS communication inequality by creating user-defined estimands. A 95% bias-corrected bootstrap confidence interval was used to test the significance levels of the effects.

## Results

### Descriptive statistics and preliminary analysis

Zero-order correlations indicated that the variables have strong positive relationships (see Table 1).

**Table 1:** Zero-order correlations, means, and standard deviations

	1	2	3	4	5	6	7
1. Urban vs. rural	1						
2. Education	.525^**^	1					
3. HIV/AIDS-related media use	.606^**^	.452^**^	1				
4. Concern about and information needs about HIV/AIDS	.639^**^	.485^**^	.479^**^	1			
5. Perceived salience of HIV/AIDS-related information	.631^**^	.316^**^	.388^**^	.398^**^	1		
6. HIV/AIDS knowledge	.654^**^	.645^**^	.504^**^	.684^**^	.464^**^	1	
7. HIV/AIDS risk perception	.561^**^	.551^**^	.549^**^	.552^**^	.401^**^	.659^**^	1
Mean	_	_	1.24	3.76	3.13	22.67	3.16
SD	_	_	.32	.76	1.05	13.14	.80

**The correlation was significant at the 0.01 level (two-tailed).

The regression analysis showed that both urbanity versus rurality (*β* = 0.47, *p* < .001) and education (*β* = 0.36, *p* < .001) were significant predictors of HIV/AIDS knowledge, which explained 57.5% of the variance (R^2^ = .56, F (4,981) = 334.11, *p <* .001) when age and gender were controlled. Similarly, both predictors (urbanity vs. rurality, *β* = 0.38, *p* < .001 and education, *β* = 0.34, *p* < .001) were significant in predicting HIV/AIDS risk perception, explaining 40.8% of the variance (R^2^ = .41, F (4,981) = 170.63, *p <* .001) when demographics were controlled.

### Testing the hypothesized Model^1^

**Figure 3:**
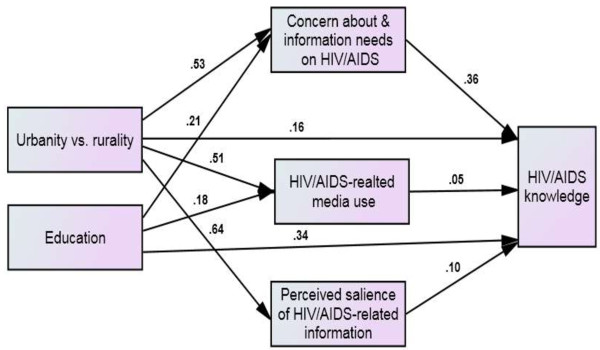
Structural model showing the relationships between urbanity vs. rurality, education, three components of communication inequality, and HIV/AIDS knowledge.

The model shown in Figure 3 estimates the hypothesized relationships between urbanity versus rurality, education, HIV/AIDS communication inequality (HIV/AIDS concern and information needs, HIV/AIDS-related media use, and the perceived salience of HIV/AIDS-related information), and HIV/AIDS knowledge. The model showed an adequate fit of the data, χ^2^(3) = 12.06, *p* = .007, CMIN/DF = 4.02, CFI = .99, RMSEA = .05, GFI = .99, AGFI = .97.

The model indicated that while urbanity versus rurality was significantly related to all three components of HIV/AIDS communication inequality (HIV/AIDS concern and information needs, *β* = 0.53, *SE* = .03, *p* = .001; HIV/AIDS-related media use, *β* = 0.51, *SE =* .03, *p* = .001; and the perceived salience of HIV/AIDS-related information, *β* = 0.64, *SE =* .03, *p* = .001), education was significantly related to only two of these components (HIV/AIDS concern and information needs, *β* = 0.21, *SE =* .03, *p* = .001; and HIV/AIDS-related media use, *β* = 0.18, *SE =* .04, *p* = .001). Moreover, all three components were found to be significantly related to HIV/AIDS knowledge: HIV/AIDS concern and information needs, *β* = 0.36, *SE* = .03, *p* = .001; HIV/AIDS-related media use, *β* = 0.05, *SE =* .02, *p* = .03; and the perceived salience of HIV/AIDS-related information, *β* = 0.10, *SE =* .03, *p* = .001.

Consistent with our prediction, the model showed that HIV/AIDS communication inequality mediated the impacts of urbanity versus rurality and education on HIV/AIDS knowledge. The total indirect effect of urbanity versus rurality through the three components of communication inequality (*β* = 0.28, *p* = .001) was stronger than that of education (*β* = 0.08, *p* = .001). Removing the insignificant path connecting education with perceived salience of HIV/AIDS-related information did indeed slightly improve the overall indirect effect of education, but the effect was still not as strong as that of urbanity versus rurality. A comparison of the fit indices of the constrained model, in which the path coefficients of the direct effects of urbanity versus rurality and education were constrained (χ^2^[5]) = 248.40, *p* = .000, CMIN/DF = 49.68, CFI = .91, RMSEA = .22, GFI = .93, and AGFI = .70) and the unconstrained model (χ^2^[3] = 12.06, *p* = .007, CMIN/DF = 4.02, CFI = .99, RMSEA = .05, GFI = .99, and AGFI = .97) further confirmed that the mediation was indeed partial; the unconstrained model yielded better fit indices than the constrained model.

When we assessed individual indirect effects, both urbanity versus rurality (*β* = 5.0, *p* = .001) and education (*β* = 1.03, *p* = .001) had their strongest effects on HIV/AIDS knowledge through HIV/AIDS concern and information needs. The second most important indirect effects of urbanity versus rurality (*β* = 1.61, *p* = .001) and education (*β* = 0.12, *p* = .02) on HIV/AIDS knowledge were through the perceived salience of the HIV/AIDS-related information and HIV/AIDS-related media use, respectively. While the least significant indirect effect of urbanity versus rurality (*β* = 0.62, *p* = .03) occurred through HIV/AIDS-related media use, education had an insignificant indirect effect (*β* = −0.03, *p* = .44) through the perceived salience of the HIV/AIDS-related information.

### Testing the hypothesized Model^2^

**Figure 4:**
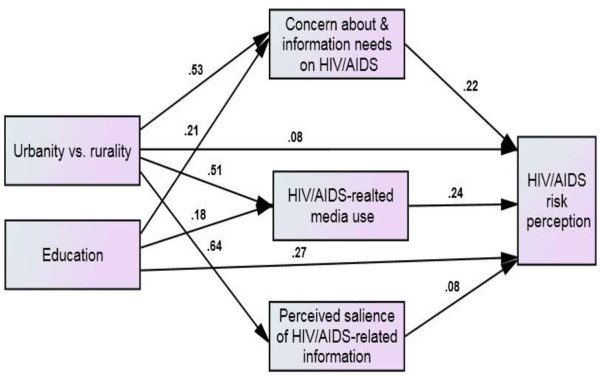
Structural model showing the relationships between urbanity vs. rurality, education, the three components of communication inequality, and HIV/AIDS risk perception.

Figure 4 shows the model with the estimates of the hypothesized relationships between urbanity versus rurality, education, HIV/AIDS communication inequality, and HIV/AIDS risk perception. The model fit indices confirmed an adequate fit of the data: χ^2^(3) = 12.06, *p* = .007, CMIN/DF = 4.02, CFI = .99, RMSEA = .05, and GFI = .99; AGFI = .97.

The effects of urbanity versus rurality and education on the three components of communication inequality were of the same direction and magnitude as those derived from Model 1. While urbanity versus rurality was significantly related to all three components of HIV/AIDS communication inequality, education was significantly related to only two of the three components. Moreover, all three of the components were significantly related to HIV/AIDS risk perception: HIV/AIDS concern and information needs (*β* = 0.22, *SE* = .03, *p* = .001; HIV/AIDS-related media use, *β* = 0.24, *SE =* .03, *p* = .001) and the perceived salience of HIV/AIDS-related information (*β* = 0.08, *SE =* .04, *p* = .02).

As with Model 1, Model 2 showed the mediating role of HIV/AIDS communication inequality. The total indirect effect of urbanity versus rurality through the three components of communication inequality (*β* = 0.30, *p* = .001) was stronger than the total indirect effect of education (*β* = 0.09, *p* = .001). Removing the insignificant path that connected education with the perceived salience of HIV/AIDS-related information slightly improved the overall indirect effect of education, but it was still weaker than that of urbanity versus rurality. Similar to Model 1, the comparison of the fit indices of the constrained model (χ^2^[5] = 112.51, *p* = .000; CMIN/DF = 22.50, CFI = .96, RMSEA = .15, GFI = .96, and AGFI = .85) and the unconstrained model (χ^2^[3] = 12.06, *p* = .007; CMIN/DF = 4.02; CFI = .99, RMSEA = .05, GFI = .99, and AGFI = .97) further confirmed that the mediation was indeed partial; the unconstrained model yielded much better fit indices than the constrained model.

Individual indirect effects showed that although urbanity versus rurality had its strongest effects (*β* = 0.20, *p* = .001) on HIV/AIDS risk perception through HIV/AIDS-related media use, education exerted equally strong effects through HIV/AIDS concern and information needs (*β* = 0.04, *p* = .001) and HIV/AIDS-related media use (*β* = 0.04, *p* = .000). The second most important indirect effect of urbanity versus rurality occurred through HIV/AIDS concern and information needs (*β* = 0.19, *p* = .001), and the weakest indirect effect occurred through the perceived salience of HIV/AIDS-related information (*β* = 0.09, *p* = .015); education had an insignificant effect (*β* = −0.001, *p* = .405) through the perceived salience of the HIV/AIDS-related information.

## Discussion

Research on the role of social factors in health has long identified the various dimensions of the social milieu that are responsible for determining health outcomes; consequently, the fact that health can be affected by various social factors is now widely acknowledged. The processes that link social factors with health outcomes are less known and remain an ongoing subject of research [3,4]. In this study, we assessed the role of communication inequality in the causal chain linking SES (education) and a socioecological factor (urbanity versus rurality) with HIV/AIDS knowledge and risk perception, drawing data from a context severely affected by the HIV/AIDS pandemic.

Informed by the SIM of health communication [15], we tested the mediating role of three instantiations of HIV/AIDS communication inequality. We found that differences in HIV/AIDS concern and information needs, HIV/AIDS-related media use and the perceived salience of HIV/AIDS-related information are some of the mechanisms through which socioeconomic and socioecological factors exert impacts on HIV/AIDS knowledge and risk perception.

Overall, the strongest effects of both urbanity versus rurality and education on HIV/AIDS knowledge and risk perception were found to be exerted through HIV/AIDS concern and information needs. The overall relationship between seeking health information and health-related outcomes has been well documented [47]. We also know from previous research, albeit mainly in the arena of cancer prevention, that paying attention to, trusting in and using health information from the media can be patterned by SES and other social factors [15]. What is less known, however, is whether this factor can at least partially mediate the link between social factors and health outcomes, particularly regarding HIV/AIDS prevention in high-prevalence areas. Therefore, the present study provides preliminary evidence that information-seeking behavior can be an important factor mediating the link between education and area of residence and HIV/AIDS knowledge and risk perception.

Apart from HIV/AIDS concern and information needs, actual HIV/AIDS-related media use has emerged as an important mediator. Consistent with previous research in other contexts and on other health problems [12], our findings suggest that media use is one of the mechanisms through which social factors, such as urbanity versus rurality and education, are more likely to exert their impacts on HIV/AIDS knowledge and risk perception. Moreover, our models suggested that the perceived salience of HIV/AIDS-related information can have an important role in mediating the impacts of urbanity versus rurality, although such a role did not hold true for education.

Our findings have several theoretical and practical implications. From a theoretical perspective, the findings suggest that media and message effects related to HIV/AIDS behavior change communication (BCC) might well be seen from a structural point of view, moving beyond the more reductionist behavioral approaches upon which most present-day HIV/AIDS communication interventions seem to be based (see Airhihenbuwa & Obregon, 2000, for the details) [48,49]. The finding that communication inequality with concomitant disparities in health-related outcomes is patterned by area of residence and education levels suggests that harnessing the media to address macro-level ecological and structural factors could be of paramount potential to deal with public health threats such as HIV/AIDS. Our observations join a growing body of research that indicates the need to move beyond individual-level factors to target larger socioecological and structural factors [50-53] by recognizing the possibility that communication events can reside at the intersection of various levels and not only one single field of influence [54,55].

The findings may also contribute to research and theories that have followed the knowledge gap hypothesis for decades [14]. Research in this line of theorizing advances the theory that the knowledge gaps between population subgroups can result from inequalities in accessing, seeking, and processing information that has been infused into a social system by the mass media [56]. One important theoretical implication of the present study could be that there is a need to revisit the decades-old international literature on the knowledge gap phenomenon and further investigate the area of HIV/AIDS behavior change communications. Indeed, as noted here and suggested by the SIM, the consequences of communication inequality are not limited to the knowledge gap phenomenon; they also span various health-related beliefs and behaviors, which indicate the need to stretch the boundaries of the knowledge gap hypothesis and reformulate its original propositions in a way that accommodates affective and behavioral outcomes and not just cognitive ones.

This study may also help to advance SIM of health communication [15]. More specifically, given that the study was conducted in a context that was not examined by the limited available studies upon which SIM rests, the findings may provide contextual breadth to the propositions put forward by the model. The study has also tested the utility of SIM for a health problem that has been minimally addressed by previous studies within SIM’s framework.

Apart from these theoretical implications, the findings may have several practical applications. First, in line with the theoretical implications that we have drawn, identifying the mechanisms through which socioeconomic and socioecological factors affect health-related outcomes could be valuable when designing prevention interventions. In line with SIM, we argue that the structural aspects of the environments (characterized by poor or no communication technology infrastructure) where the rural populace and people with low education levels live and the social roles that these people inhabit may place them in positions that prevent them from benefiting from ongoing HIV/AIDS communication efforts compared with their urban and better-educated counterparts. This disparity may require a re-examination of our tacit assumptions that communication interventions based on the use of mass media can be used to address the “masses” across the board. More specifically, communication interventions may need to identify the communication needs of disadvantaged groups (the rural populace and people with low education levels, in this case) to move beyond the traditional biases of generalizing effects.

Second, and more specifically, our findings indicated that the rural populace and people with low education levels seem to be unconcerned or have little concern about HIV/AIDS; consequently, their overall use of the mass media to obtain prevention information was relatively low. Therefore, BCC efforts in the region might need to alert the poorly educated rural populace about the danger of infection.

Third, research has shown that concern about a given health problem affects the effectiveness of the prevention messages about that health problem [57]. Specifically, research in the area of safer sex has documented that individuals were more likely to engage in preventive behaviors (such as condom use) to the extent that their search for sexual health information increased [58]. Hence, to obtain the desired health outcomes across population subgroups (i.e., urban and rural residents and socioeconomic subgroups), BCC efforts must first address the differences in these groups’ concern about and information needs regarding the pandemic. Moreover, the differences in concern may require using different theoretical models to plan BCC interventions. The elaboration likelihood model, for example, stresses the need to match the message format to the recipients’ level of concern, explicating two different routes of persuasion for unconcerned and concerned audiences [59]. For example, a much more direct and stronger message might be more fitting for urbanites and those with better educations, while a softer and more entertainment-oriented message might be suitable for rural residents and those with low education levels.

Finally, our findings suggest that low levels of perceived salience of HIV/AIDS-related information among the rural populace might have contributed to their reduced levels of HIV/AIDS-related media use. The rural populace could thus be motivated to use the mass media for HIV/AIDS information if the content was more relevant and appealing to them. In practice, this would mean that programmatic adjustments must be made in such a way that mass media HIV/AIDS campaigns could include contents more relevant to the rural populace while also providing messages that are relevant to urbanites. Indeed, it is conceivable that there could be various other factors that contribute to the overall lack of HIV/AIDS-related media use among the rural populace and the low SES population sub-group. For instance, previous research in sub-Saharan Africa has suggested that non-HIV life expectancy, which could vary by SES and area of residence, could be one of the factors importantly related to behavioral responses to the infection [60]. It could therefore be hypothesized that population sub-groups with limited non-HIV life expectancy might be inclined to ignore the danger of the infection and any prevention information associated with it. Clearly, future research needs to pursue such intriguing findings.

### Limitations

As any study, the merits of the present paper should be evaluated based on a contributions-to-limitations assessment [61]. The study has several limitations that need to be taken into account in drawing generalizations based on the findings. First, although our findings are partially supported by previous studies that have drawn data from multiple sub-Saharan African countries [62], the limited nature of our data should be noted. Second, in most studies dealing with causation, the effect of a confounding factor is a real problem. As such, despite the use of rigorous statistical techniques and considerable attempts of controlling for demographic variables such as age and gender, the fact that the causal chain between the exogenous and endogenous variables may have been colored by other extraneous variables cannot be totally ruled out. Moreover, the fact that we used brief measures for some of our variables should be taken into account, although internal consistency tests have been performed for each variable. Indeed, the advantages of such short measures for reducing confusion and response burden among low literate respondents should equally be borne in mind. Due mainly to time and resource constraints, the study did not utilize several socioeconomic and socioecological indicators, and relied on two prominent indicators that are commonly taken as reliable proxies. These limitations imply the need for further research on the issue by taking the findings of the present study as tentative but important first steps that have provided evidence of the role communication inequality plays in the realm of HIV/AIDS prevention.

## Conclusion

Because we live in an era whose main distinctive feature is arguably the increased vitality of information and communication, the control of information is power, and people and/or population subgroups with the capacity to generate, access, use, and redistribute information clearly possess the social power and the privileges that accrue from it [14,15,63]. The implication of this theory is so important that if disparities in HIV/AIDS-related outcomes are to be tackled, the communication inequalities across population subgroups must be counteracted. Overall, the role of communication inequality, which was discerned in the present study, further reaffirmed the notion that BCC must remain the mainstay of the global fight against HIV/AIDS. However, communication interventions may need to refocus on larger structural and socioecological factors to augment the promising results that have been obtained to date regarding the individual-level cognitive, affective, and behavioral outcomes across intervention contexts [64,65].

## Competing interests

The authors declare that they have no competing interests.

## Authors’ contributions

Both authors contributed to the manuscript equally. Both authors conceived and designed the study. MAB collected, analyzed and interpreted the data. SE contributed to the analysis and interpretation of the data. MAB drafted and revised the manuscript. SE was involved in drafting and revising the manuscript. Both have given final approval of the version to be published.

## Authors’ information

MAB is a doctoral student at the University of Leuven, Belgium. He is affiliated to Bahir Dar University, Ethiopia. His research focuses on behavior change communication. He is currently at the Harvard School of Public Health to finalize his doctoral research. SE is an associate professor at the University of Leuven. He is the director of the communication sciences program at the faculty of social sciences, University of Leuven.
